# Mobile Phone Apps in Australia for Improving Pregnancy Outcomes: Systematic Search on App Stores

**DOI:** 10.2196/22340

**Published:** 2020-11-16

**Authors:** Loretta M Musgrave, Nathalie V Kizirian, Caroline S E Homer, Adrienne Gordon

**Affiliations:** 1 Centre for Midwifery, Child and Family Health University of Technology Sydney Ultimo NSW Australia; 2 Faculty of Medicine and Health Charles Perkins Centre University of Sydney Camperdown NSW Australia; 3 Burnet Institute Melbourne VIC Australia; 4 Centre for Midwifery, Child, and Family Health University of Technology Sydney Ultimo NSW Australia; 5 Sydney Local Health District NSW Health Camperdown NSW Australia

**Keywords:** smartphone apps, mobile phone, pregnancy, health behavior change, MARS tool, CALO-RE taxonomy, pregnancy outcomes, quality assessment methods

## Abstract

**Background:**

Women are increasingly turning to mobile health platforms to receive health information and support in pregnancy, yet the content of these platforms vary. Although there is great potential to influence health behaviors, little research has assessed the quality of these platforms or their ability to change behavior. In recent years, validated tools to assess app quality have become available.

**Objective:**

To identify and assess the quality and ongoing popularity of the top 10 freely available pregnancy apps in Australia using validated tools.

**Methods:**

A systematic search on app stores to identify apps was performed. A Google Play search used subject terms pregnancy, parenting, and childbirth; the iTunes search used alternative categories medical and health and fitness. The top 250 apps from each store were cross-referenced, and the top 100 found in both Google Play and iTunes were screened for eligibility. Apps that provided health information or advice for pregnancy were included. Excluded apps focused on nonhealth information (eg, baby names). The top 10 pregnancy apps were assessed using the Mobile App Rating Scale (MARS). A comparative analysis was conducted at 2 time points over 2 years to assess the ongoing popularity of the apps. The MARS score was compared to the download and star rating data collected from iTunes and Google Play in 2017 and 2019. Health behaviors including breastfeeding, healthy pregnancy weight, and maternal awareness of fetal movements were reviewed for apparent impact on the user’s knowledge, attitudes, and behavior change intentions using the MARS perceived impact section and the Coventry, Aberdeen, and London—Refined (CALO-RE) taxonomy.

**Results:**

A total of 2052 free apps were screened for eligibility, 1397 were excluded, and 655 were reviewed and scored. The top 10 apps were selected using download numbers and star ratings. All 10 apps were suboptimal in quality, practicality, and functionality. It was not possible to identify a primary purpose for all apps, and there was overlap in purpose for many. The mean overall MARS app quality score across all 10 apps was 3.01 (range 1.97-4.40) in 2017 and 3.40 (range 2.27-4.44) in 2019. A minority of apps scored well for perceived impact on health behavior using the MARS tool. Using the CALO-RE 40 item taxonomy, the number of behavior change techniques used was low. The mean number of behavior change techniques for breastfeeding was 5 (range 2-11), for pregnancy weight was 4 (range 2-12), and for maternal awareness of fetal movements was 5 (range 2-8).

**Conclusions:**

This review provides valuable information to clinicians and consumers about the quality of apps currently available for pregnancy in Australia. Consideration is needed regarding the regulation of information and the potential opportunity to incorporate behavior change techniques to improve maternal and fetal outcomes.

## Introduction

Smartphone ownership and app use in Australia are high, with 81% of people possessing a smartphone, and 97% of mobile consumers aged between 18 and 34 years [1]. In 2019, mobile phones were the most common device used to access the internet (87%), followed by a laptop (69%), then tablets (56%) [2]. The most recent Australian data suggest that 46% of internet users access the internet for health services; this is an increase from 22% in 2014-2015 [3]. It has been estimated that up to 1 in 4 Australians use their smartphones to access health-related apps to support healthy behaviors [4].

Pregnant women are increasingly turning to mobile health (mHealth) to receive health information and support rather than relying on face-to-face and paper-based delivery methods [5-11]. This use of mobile health apps during pregnancy provides a unique window of opportunity—a teachable moment—when women are often more motivated to optimize health and change their lifestyle [12,13]. Apps also have the potential to act as a platform for specific pregnancy behavior change interventions, such as maternal awareness of decreased fetal movements, maternal weight monitoring, and breastfeeding [14-17]. A recent systematic review [18] found limited data of the effects of mobile app interventions during pregnancy on maternal knowledge and behavior change. This review [18] concluded that well-designed studies are needed to evaluate apps. App developers should include women in co-design, implementation, and evaluation phases of development. This was further supported by a systematic review [19] that aimed to evaluate usability (feasibility and acceptability) as well as the effectiveness of lifestyle and medical apps in supporting health care during pregnancy in high-income countries. The review [19] concluded that further evidence is needed before such apps are implemented in health care. For apps to be used as an adjunct to health care, issues related to the accuracy of the information, privacy, and security also need to be addressed [19].

Health behaviors such as maternal awareness of decreased fetal movements, maintaining a healthy weight in pregnancy, and breastfeeding are modifiable behaviors with known benefits for both mothers and babies. Globally, apps have been used to address such behaviors; however, further evidence is needed to establish if these apps have an impact on pregnancy outcomes [18]. In Australia, such apps have been assessed in a research setting, including Growing Healthy, which provides information on healthy infant feeding [20], and the My Baby’s Movements app, which provides information about normal fetal movements and has a tracking tool [21]. In 2018, a quasi-experimental study [20] was conducted to describe the effects of Growing Healthy on parental feeding practices, infant food preferences, and infant satiety responsiveness; the authors concluded that mHealth design and delivery characteristics that impact on infant feeding practices need further research. My Baby’s Movements has also been tested in a randomized controlled trial [21]; results are not yet published.

Women who are overweight or obese have an increased risk of pregnancy complications [22]. Pregnancy weight gain can be addressed through lifestyle and dietary interventions [23]. Institute of Medicine weight gain recommendations [24], National Institute for Health and Clinical Excellence guidelines [25], and Australian dietary and physical activity guidelines are referenced and recommended in national Clinical Practice Guidelines for pregnancy care [26]. A recent systematic review of nutritional information available to pregnant women on smartphones in the United Kingdom found that apps do not consistently provide useful or accurate nutritional information [27].

The health benefits of exclusive breastfeeding for 6 months for mothers and infants are well established [28]. A 2019 systematic review [29] of digital interventions that support breastfeeding found that there is potential to improve breastfeeding outcomes. A recent cohort study [30] conducted in the United Kingdom evaluated the effectiveness of the Baby Buddy app; Baby Buddy is an mHealth intervention that is available on the UK National Health Service Library that supports and guides women through pregnancy and the first 6 months of their child’s life. The posthoc analysis of this study suggested that Baby Buddy app users were more likely to report exclusively breastfeeding or ever breastfeeding [30].

This study aimed to identify and review the top 10 pregnancy apps available in Australia over 2 years using validated tools to assess the quality and perceived impact of 3 important pregnancy health behaviors.

## Methods

### Study Design

This review used a stepwise systematic approach to identify, select, assess, and evaluate the 10 most popular pregnancy apps in Australia from November 2017 to October 2019. We assessed their quality and use of behavior change techniques for 3 specified behaviors— maternal awareness of decreased fetal movement, managing weight in pregnancy, and breastfeeding—using validated tools [31,32].

### Step 1: Selection of Smartphone Apps

Apps were identified using a search strategy developed using PRISMA-P (Preferred Reporting Items for Systematic Reviews and Meta-Analysis Protocol) guidelines [33] for reporting systematic reviews evaluating health care interventions. Both iTunes (Apple Inc, Australia) and Google Play (Google Inc, Australia) were searched using a set of terms developed for each online app store. The searches were conducted on the authors’ smartphones. Google Play search terms included *pregnancy*, *parenting*, and *childbirth*. Categories searched in iTunes were *medical* and *health and fitness*. The top 250 apps from each store that met the search terms were cross-referenced to find the top 100 available in both Google Play and iTunes. These 100 apps were then screened for eligibility. Apps were screened for relevance based on the inclusion criteria, using the information provided in the app store description. The top 10 apps were then selected using the download numbers and star ratings provided in each store. A comparative analysis of the top 10 apps was conducted at 2 time points, in November 2017 and in October 2019. Discrepancies regarding the selected apps were discussed and resolved by the review team.

Apps were included in the search that satisfied the following criteria: available free (with or without in-app purchase) AND modifiable or interactive AND provided general pregnancy information or education. In addition, there was a criterion that the app either aimed to support targeted behavior change in pregnancy such as healthy diet and exercise or aimed to support general well-being and disease prevention in pregnancy including mental health. Apps were excluded in the search if they satisfied any of the following criteria: a cost was involved, the app was not available in both Australia iTunes or Google Play stores, the app was not available in English, the app was not designed for interactive use, the app was primarily designed to track or assist with contraception or fertility, the app was classified as a game or entertainment only and was not designed for education or information delivery (eg, baby names), or the app was designed primarily for use by other consumers (such as health care professionals, women’s partners).

### Step 2: Evaluation of Smartphone Apps

#### App Classification and Quality

Two reviewers classified and evaluated the quality of the top 10 pregnancy apps using the Mobile Application Rating Scale (MARS) [31]. The MARS tool was chosen as it has proven reliability through test-retest studies and has excellent internal consistency [31]. MARS has been validated for health applications and has been used in several studies, for example, pregnancy-specific nutrition apps [34,35], medication adherence [36], apps for treatment of speech disorders in children [37] and pain management [38]. Using the descriptive information provided by each app, the reviewers identified the focus and theoretical background (or strategies) used by the app developers. Affiliations, technical aspects, and target age groups were also examined. The 23-item tool has 4 objective quality subscales and 1 subjective quality rating scale. A 5-point rating scale (1, inadequate, to 5, excellent) was used for each of the 4 objective subscales: engagement, functionality, aesthetics, and information quality. A mean score was calculated that ranged from 1-5. A score of 5 denoted excellent quality, and a score of 1 indicated poor app quality [31]. The overall app quality score was calculated using the scores for the 4 domains. MARS has been designed in this way so that the total score can be directly translated to a star rating, and therefore, can be easily compared with app stores. Each app was assessed for subjective quality using a 5-point scale and calculated mean. The 4 subjective items were potential benefit, use, cost, and overall personal star rating (a score of 5 denoted “One of the best apps I’ve used” and a score of 1 denoted “One of the worst apps I’ve used”) [31]. Reviewers used each app for at least 10 minutes and assessed how easy the app was to use. The 10 included apps were assessed on both iOS and Android devices to determine if there was any variance in functionality or usability between the different platforms. Data related to app settings, developer information (affiliations), external links, and security features were also reviewed.

#### Comparative Analysis

At 2 time points, 2 years apart, a comparative analysis of the MARS scores, downloads and star ratings of the top 10 apps was conducted to assess the ongoing quality and popularity of the selected apps. Data were collected from iTunes and Google Play on November 3, 2017 and October 5, 2019. This was done to assess whether the quality or download rating had changed.

### Step 3: Analysis of Behavior Change Techniques Used

The 3 prespecified target health behaviors were found in all 10 apps. These behaviors were assessed in 2019 using both the additional component of the MARS tool [31], perceived impact, and the Coventry, Aberdeen, and London—Refined (CALO-RE) taxonomy [32]. This was undertaken to compare which apps could be effective in modifying behavior change. Content related to the behaviors was reviewed in each app to assess potential impact on user awareness, knowledge, attitudes, intentions to change, help-seeking, and behavior change and was documented using a 5-point rating scale from 1 (strongly disagree) to 5 (strongly agree) [31]. To assess the perceived impact, we used a method described by Furlong et al [37]. We identified best-practice principles for decreased fetal movement awareness, weight management, and breastfeeding as well as the intervention techniques used by the apps (eg, self-monitoring, instruction on how to perform the behavior, and information on consequences of behavior). A mean score was then calculated [37]. The CALO-RE tool was chosen as an adjunct to MARS perceived impact as it has been used successfully for reporting, evaluating, and implementing physical activity, healthy eating, and lifestyle interventions [34]. CALO-RE is a systematic way to apply evidence and theory linked to behavior change using a taxonomy consisting of 40 behavior change techniques items [32]. Each app was reviewed for all 3 target behaviors. Definitions were used to accurately describe the behavior change techniques such as goal setting, action planning, and barrier identification [32]. Using a method described by Brown et al [34], we assessed the frequency of behavior change technique inclusion in all 3 behaviors in all 10 apps. To do this, we reviewed app content, assigned individual behavior change techniques as defined by Michie et al [32] and calculated a score out of 40 possible behavior change techniques [34]. We repeated this process for each behavior.

### Fetal Movement Awareness

Information about fetal well-being and advice given regarding decreased fetal movement were examined using the Australian Safer Baby Bundle Handbook and Resource Guide [39]. Using MARS and CALO-RE, we considered the potential impact that each app would have on changing women behaviors toward monitoring fetal movements and the likelihood that they would act on concerns and contact a health care provider.

### Healthy Weight in Pregnancy

Advice on gestational weight gain included in apps was reviewed against US Institute of Medicine weight gain recommendations, the UK National Institute for Health and Clinical Excellence weight management guidelines for pregnancy, and the Australian clinical practice guidelines for pregnancy care.

### Breastfeeding

Breastfeeding content was compared for alignment with the World Health Organization (WHO) Breastfeeding Friendly Hospital Initiative 10 Steps to Successful Breastfeeding [40] and International Code of Marketing of Breast-Milk Substitutes [41]. Information, images, advertising, and sponsorship of each app were reviewed and evidence of breaches of the WHO Code was collated alongside the MARS and CALO-RE data.

## Results

### Step 1: Selection of Smartphone Apps

**Figure 1:**
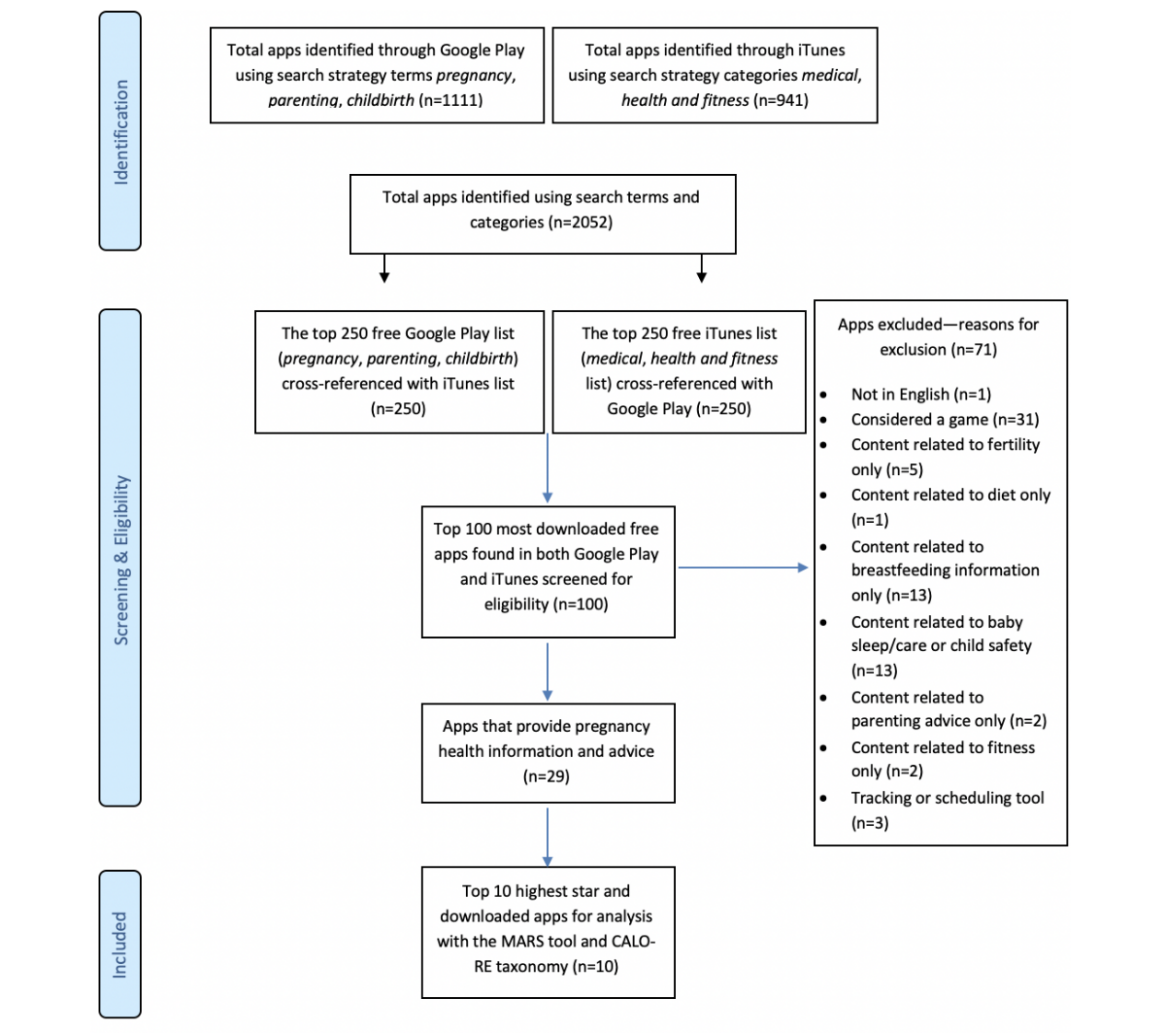
Flowchart of the app selection process.

In November 2017, a total of 2052 apps were identified. Of these, 1111 apps were found only in Google Play, and 941 were found only in iTunes. Due to the volume of apps, the top 250 free apps in both stores were cross-referenced to identify the top 100 most downloaded. A total of 71 apps were excluded, and 29 met inclusion criteria. Of these 29 apps, the 10 apps with the highest number of downloads and star ratings in both stores were identified (Figure 1). The following apps were assessed for quality: Ovia Pregnancy Tracker, I’m Expecting, Baby Centre, Pregnancy +, Glow, What to Expect, Baby Bump Pregnancy Pro, Sprout Pregnancy, Week by Week, The Bump. In 2019, the top 10 apps identified from 2017 were again searched for in both stores to conduct a comparative analysis of download and star ratings at 2 time points.

### Step 2: Evaluation of Selected Smartphone Apps

#### App Classification

The focus, theoretical background, and strategies used in each app were difficult to ascertain. Affiliations and sources of funding information available indicated that all 10 were commercially developed. All apps lacked transparency regarding the details of funding. The review team was unable to determine the target age groups as these were not specified. The app description, design, functionality, and content were designed to appeal to women of reproductive age; however, some had user pathways for partners and carers. Of the 10 apps, 7 had reminders, 7 allowed password protection, 7 had an app community, 9 allowed sharing, 9 required logins, and all 10 required web access.

There was no single dedicated focus described for any of the 10 apps, and there was an overlap between categories. Four of the apps had additional categories that were not prespecified (other), these linked directly to online stores to upgrade the app or buy baby-related products (4/10 apps). The entertainment category included apps that had links to online communities and information that was not always related to pregnancy health, and well-being. The physical health category included apps that provided information about pregnancy symptoms, milestones, nutrition, exercise, maternal weight gain, medication, and prenatal vitamin reminders (10/10 apps). Of the 10 apps, 5 had a component that related to supporting mental health (reducing negative feelings, anxiety, and depression).

All 10 apps provided some health information and education. All app descriptions stated that the app would help monitor and track various healthy behaviors, provide advice, tips, and strategies; 9 out of 10 apps had such content. Further analysis of the content using the MARS tool showed that few provided the necessary goal-setting (4/10 apps), assessment (3/10 apps), and feedback (2/10 apps) required to successfully support behavior change.

#### App Quality

The mean of the 4 MARS subscale scores across all 10 apps were 3.01 (range 1.97-4.40) in 2017 and 3.40 (range 2.27-4.44) in 2019. The app that had the highest quality scores was Ovia Pregnancy Tracker App (in 2017: mean 4.40; in 2019: mean 4.44). The app that had the lowest quality score in 2017 (mean 1.97) was Baby Bump Pregnancy Pro. This app was no longer available for download in 2019, therefore, was not analyzed at the second time point. In both 2017 and 2019, functionality was rated the highest in both 2017 and 2019 followed by aesthetics, engagement, and information (Table 1). Ovia Pregnancy Tracker scored highest across all subscales. Apps that scored higher for engagement and aesthetics scored lower for information. We found that sources of information were not cited, and studies and trials were not included. There was inconsistent information, and there appeared to be an ad hoc approach with several pieces of content missing review dates. The subjective quality items were calculated as a mean score. Apps that had the highest subjective scores in 2017 were Ovia Pregnancy Tracker (mean 3.75) and Sprout Pregnancy (mean 2.75). These 2 apps also had the highest overall MARS scores in 2017 (mean 4.40 and mean 3.38, respectively).

**Table 1:** MARS scores of the Top 10 apps in 2017 and 2019.

App	Engagement score		Functionality score		Aesthetics score			Information score			Overall quality score		
	2017	2019	2017	2019		2017	2019		2017	2019		2017	2019
All, mean	3.00	3.40	3.62	4.25		3.30	4.00		2.10	2.14		3.01	3.40
Ovia Pregnancy Tracker	4.60	4.60	5.00	4.75		5.00	5.00		3.00	3.42		4.40	4.44
I’m Expecting	3.00	2.20	3.50	3.00		3.00	2.66		2.11	2.00		2.90	2.46
Baby Centre	3.00	3.60	3.00	4.50		3.00	5.00		2.70	2.85		2.90	3.98
Pregnancy +	3.00	3.40	3.75	4.25		4.10	4.33		1.70	2.22		3.11	3.55
Glow	3.40	3.00	4.00	4.33		3.30	3.66		2.10	2.14		3.20	3.28
What to Expect	3.00	4.00	3.50	4.50		3.30	4.66		1.42	2.42		2.80	3.89
Baby Bump Pregnancy Pro	2.20	—^a^	2.20	—		2.30	—		1.20	—		1.97	—
Sprout Pregnancy	3.00	2.60	4.00	2.25		4.33	2.66		3.00	1.57		3.58	2.27
Week by Week	2.20	2.80	4.00	3.50		4.00	3.33		2.28	2.14		3.12	2.84
The Bump	2.00	4.20	2.75	4.00		3.00	4.00		1.40	1.42		2.28	3.40

^a^Denotes that this app was not available for download in 2019.

#### Comparative Analysis

Baby Bump Pregnancy Pro had the lowest MARS score in 2017 and was not available to download at the second time point on either iOS or Android. For the majority of apps, there was an increase in the mean quality scores from 2017 to 2019. The exceptions to this were I’m Expecting, Sprout Pregnancy, and Week by Week, which had lower scores than their 2017 scores. Sprout Pregnancy had the lowest star rating (4.5) at both time points. This app dropped significantly in ranking from 2017 (ranked second) to 2019 (ranked ninth). Ovia Pregnancy Tracker ranked first at both time points (Table 2).

**Table 2:** Comparison of Android downloads (rounded estimates as shown in Google Play) and star ratings for the top 10 apps.

App	Downloads		Star ratings		
	2017	2019		2017	2019
Ovia Pregnancy Tracker	81,053	1,000,000		4.8	4.8
I’m Expecting	70,640	1,000,000		4.6	4.6
Baby Centre	530,321	10,000,000		4.7	4.7
Pregnancy +	157,385	10,000,000		4.5	4.6
Glow	16,938	500,000		4.6	4.6
What to Expect	43,882	1,000,000		4.5	4.6
Baby Bump Pregnancy Pro	26,659	—^a^		4.5	—
Sprout Pregnancy	23,131	1,000,000		4.5	4.5
Week by Week	1100	1,000,000		4.8	4.9
The Bump	13,532	1,000,000		4.5	4.7

^a^Denotes that this app was not available for download in 2019.

### Step 3: Analysis of Behavior Change Techniques Used

Overall, Ovia Pregnancy Tracker scored the highest for perceived impact using the MARS tool across all 3 behaviors (breastfeeding: mean 3.0; healthy weight: mean 3.5; maternal fetal movement awareness: mean 4.0; all apps: range 1-4). When examined using CALO-RE, Ovia Pregnancy Tracker did not have the highest number of behavior change techniques for any of the behaviors reviewed (Table 3). What to Expect used the highest number of behavior change techniques (breastfeeding: 11/40; healthy weight: 9/40; maternal fetal movement awareness: 8/40; all apps: range 2-12) (Table 3). A detailed analysis of the frequency of CALO-RE behavior change techniques included across the 10 apps for the 3 behaviors is included in Multimedia Appendix 1.

### Fetal Movements

Ovia Pregnancy Tracker scored the highest for perceived impact for fetal movements (mean 4.0; range 1-4) (Table 3). When assessed using CALO-RE, the highest number of behavior change techniques used was 8/40 (range 2-8). When cross-referenced with the Australian Safer Baby Bundle Handbook and Resource Guide [39], we found several discrepancies. All apps had some inaccurate or incomplete information about maternal fetal movement monitoring. One app stated that normal baby movement was 10 kicks in 2 hours and all other apps provide no or partial information alongside the in-app tools provided. Three of the 10 apps incorrectly suggested the mother should consume something sweet to encourage fetal movements. One app recommended buying a fetal Doppler ultrasound, claiming that it was beneficial and so that the pregnant woman could monitor fetal well-being. This is not evidence-based and may impact negatively on fetal outcomes [42]. Three of the apps did not articulate or encourage women to contact a health care provider if concerned about decreased fetal movements or mention the risk of stillbirth.

### Healthy Weight in Pregnancy

Ovia Pregnancy Tracker scored the highest for perceived impact (mean 3.5, range 1.0-3.5). Basic information on diet and exercise was included in all apps, with a focus primarily on fitness rather than weight management. Tools to track exercise and weight were included in 5 of the apps; however, little or no information was given on how, when, or why it is important to do so. Weight tracking tools in 2 apps provided incorrect information on expected weight gain, and 2 apps provided information that was misleading regarding increasing calorie intake and advocating the need to eat for two. CALO-RE analysis showed that Baby Centre (12 techniques) and What to Expect (9 techniques) utilized the highest of behavior change techniques (all apps: mean 4.4, range: 2-12), but there was no clear alignment with Institute of Medicine, National Institute for Health and Clinical Excellence, or Australian pregnancy care guidelines (Table 3).

### Breastfeeding

Basic breastfeeding information was provided in all apps; however, the content did not adequately cover all aspects of breastfeeding, was inaccurate, or did not follow best practice outlined in the WHO code [41]. Although Ovia Pregnancy Tracker scored the highest for perceived impact (mean 3.0), it had one of the lowest numbers of behavior change techniques (5/40) when compared against other apps (What to Expect: 11/40; The Bump: 10/40). Two apps provided information for later in pregnancy and highlighted some difficulties with breastfeeding. It was noted that these apps did not mention midwives or lactation consultants as a form of support, instead suggesting that formula is equal to breastmilk. The apps varied greatly; 1 app provided a feeding tracker and gave links to relevant articles while another app gave information that was directly linked to online shopping for nipple creams and bras. One app had affiliations with a company that sells breastmilk substitutes; this app scored the lowest in both MARS and CALO-RE and directly contradicted the WHO code [41].

**Table 3:** Perceived impact of app on modifiable healthy behavior using the MARS app-specific tool and CALO-RE behavior change techniques taxonomy.

App	Breastfeeding			Healthy weight			Fetal movements	
	MARS^a^ perceived impact score, mean	CALO-RE^b^ behavior change techniques, n (%)	MARS perceived impact score, mean		CALO-RE behavior change techniques, n (%)	MARS perceived impact score, mean		CALO-RE behavior change techniques, n (%)
Ovia Pregnancy Tracker	3.0	5 (12.5)	3.5		4 (10)	4.0		4 (10)
I’m Expecting	1.1	2 (5)	1.6		2 (5)	1.6		5 (12.5)
Baby Centre	1.1	9 (22.5)	1.3		12 (30)	1.3		7 (17.5)
Pregnancy +	1.0	6 (15)	3.0		3 (7.5)	3.0		7 (17.5)
Glow	2.5	2 (5)	1.8		4 (10)	1.0		3 (7.5)
What to Expect	3.0	11 (27.5)	2.3		9 (22.5)	1.5		8 (20)
Baby Bump Pregnancy Pro	1.0	—^c^	1.3		—	1.3		—
Sprout Pregnancy	1.0	2 (5)	1.5		2 (5)	2.3		2 (5)
Week by Week	1.0	2 (5)	2.3		2 (5)	2.3		8 (20)
The Bump	1.0	10 (25)	1.0		2 (5)	1.0		8 (20)

^a^MARS: Mobile Application Rating Scale.

^b^CALO-RE: Coventry, Aberdeen, and London—Refined.

^c^Denotes that this app was not available for download in 2019.

## Discussion

### Principal Findings

This review showed that highly rated pregnancy smartphone apps were generally of low to moderate quality. This study is the first to have systematically described app quality and the use of behavior change techniques in pregnancy apps for 3 key health behaviors—maternal fetal movement monitoring, pregnancy weight monitoring, and breastfeeding.

The trend toward the use of mobile health opens up an opportunity to reach women who are less likely to or have yet to engage with health care providers. The information provided in all 10 apps, however, was not tailored for specific groups, for example, young mothers. We were unable to find any information in the 10 apps that suggested that pregnant women were engaged as co-designers at any stage of the app development or that endorsement from key maternity care organizations, health departments, or colleges was sought. All apps required web access. This may be a barrier for women living outside major Australian cities with limited Wi-Fi connection or with restricted data plans. Those who may benefit most are potentially not able to access interventions that may improve health outcomes and support pregnancy and early parenthood.

Functionality appears to be the main focus for developers. All 10 apps work technically well and have some in-built mechanisms for sharing, basic privacy settings (to meet Australian law), and rudimentary personalization capability. All reviewed apps lacked transparency regarding affiliations and have been set up to be commercial rather than as an intervention to change behavior. Although the functionality and usability of the apps have increased in the last two years, content credibility has not. Several reviews have found that health apps have insufficient evidence-based content [43-45]. Since there is limited regulatory oversight of the quality of apps and content provided, the MARS tool could be used by clinicians and by women themselves to identify high-quality apps rather than relying on download and star ratings. Our results confirm that few apps provide evidence-based information; therefore, caution is advised before recommending the use of these during pregnancy. For example, regarding maternal fetal movement awareness, reviewed app tools appeared to be for entertainment purposes since they were designed poorly and lacked essential information. We were unable to determine if fetal movement tools in apps during pregnancy would positively impact maternal knowledge, behavior change, or perinatal health outcomes. Of concern is the possibility of women assuming that app content is evidence-based and credible; however, in reality, apps are a platform for in-app purchases and link to unnecessary and potentially harmful advice. This was highlighted in the breastfeeding information with links to breastmilk substitutes and pictures of idealized artificial feeding. Such breaches of the WHO code do not contribute to motivating women to breastfeed exclusively for 6 months or seek help and assistance to do so.

### Strengths and Limitations

The strength of this study is the use of a stepwise systematic approach to identify and review pregnancy mobile apps in Australia. By assessing current apps using the MARS tool and CALO-RE taxonomy, we were able to evaluate features and quality, as well as capture the usefulness of these apps as behavior change intervention strategies. We looked at the top 10 apps in terms of popularity using downloads and star ratings in Australian iOS and Android app stores. We then assessed 3 prespecified healthy pregnancy behaviors. Our inclusion criteria were deliberately set wide to assess what apps are commonly used by pregnant women; the disadvantage of using this criteria was that we may have missed other behaviors.

Our study supports the work by Brown et al who reviewed the quality of Australian iPhone pregnancy apps and the inclusion of behavior change techniques for pregnancy-specific nutrition information using MARS and CALO-RE [34] and free pregnancy apps available in the Google store [35]. Similarly, we found that only a small number of behavior change techniques were utilized across the 3 healthy behaviors we examined using CALO-RE. Likewise, our MARS findings had an overall mean quality score of 3.02 compared to the mean of 3.05 in Brown et al [34]. We found no single tool could cover all aspects of pregnancy apps and therefore we used both the MARS tool and the CALO-RE taxonomy. We chose the MARS tool as it has been validated for the quality of health apps. CALO-RE was used because it has more detail about specific behavior change techniques.

CALO-RE and perceived impact scores in this study are low. This may be attributed to the lack of behavior change theory in app design or that behavior change was not the purpose of the apps. The number of behavior change techniques used and the perceived impact does not necessarily correlate with actual behavior change as a result of using these apps. A feasibility study would need to be conducted to establish the link between perceived and actual impact, this is beyond the scope of this study; however, using CALO-RE and the MARS tool to assess the likelihood of behavior change has highlighted the need for improvement if apps are to be used as interventions.

Finally, searching for pregnancy apps is problematic due to inconsistent search terms across iTunes and Google Play. The implication of this, is that a search cannot be replicated, and therefore, validated. A potential solution to facilitate searching would be the development of a vocabulary for app indexing similar to Medical Subject Headings [46]. This would enable users and researchers alike, to have the ability to easily find the most appropriate apps with pregnancy information. Also, with the constant addition and removal of apps from the market, it is difficult to provide a timely appraisal of the current apps available. Future research could explore the creation of a combined scoring tool for pregnancy apps that could be used by both clinicians and women.

### Conclusions

This study confirms that publicly available, free pregnancy apps in Australia should be treated with caution rather than recommended. Clinicians and researchers need to work collaboratively and show leadership in developing evidence-based pregnancy apps that incorporate behavior change techniques. Engagement with pregnant women in co-design must occur at all stages of app development, and endorsement from peak maternity care organizations, health departments, and professional societies should be sought. Smartphone apps have the potential to influence healthy behaviors in pregnancy, but an evidence-based approach is needed.

## Appendix

Multimedia Appendix 1 Frequency of behavior change technique inclusion.

## Abbreviations

CALO-RE: Coventry, Aberdeen, and London—Refined
MARS: Mobile Application Rating Scale
mHealth: mobile health
WHO: World Health Organization
