# Supplementary material for: Mobile Phone Apps in Australia for Improving Pregnancy Outcomes: Systematic Search on App Stores
Source: JMIR Mhealth Uhealth. 2020 Nov 16;8(11):e22340. doi: 10.2196/22340 (PMC7704277; doi:10.2196/22340)
Supplement: Multimedia Appendix 1 [file mhealth_v8i11e22340_app1.docx]

Appendix 1

Frequency of behaviour change technique inclusion across the apps (Breastfeeding n = 49. Maternal fetal movement monitoring n = 52, Healthy weight in pregnancy n = 40)

| Behaviour change technique (BCT) | Breastfeeding - Number of apps that included the BCT (%) | Maternal fetal movement monitoring – Number of apps that included the BCT (%) | Healthy weight in pregnancy – Number of apps that included the BCT (%) |
| --- | --- | --- | --- |
| 1. Provide information on consequences of behaviour in general | 9 (18) | 9 (17) | 9 (22) |
| 2. Provide information on consequences of behaviour to the individual | 8 (16) | 3 (5) | 5 (12) |
| 4. Provide normative information about others’ behaviour | 3 (6) | 3 (5) | 4 (10) |
| 5. Goal setting (behaviour) | 0 (0) | 5 (9) | 2 (5) |
| 6. Goal setting (outcome) | 5 (10) | 4 (7) | 5 (12) |
| 8. Barrier identification/problem solving | 2 (4) | 0 (0) | 2 (5) |
| 15.Prompting generalisation of a target behaviour | 4 (8) | 4 (7) | 1 (2) |
| 16. Prompt self-monitoring of behavioural | 1 (2) | 3 (5) | 2 (5) |
| 17. Prompt self-monitoring of behavioural outcome | 0 (0) | 2 (3) | 3 (7) |
| 18. Prompting focus on past success | 1 (2) | 0 (0) | 1 (2) |
| 20. Provide information on where and when to perform the behaviour | 3 (6) | 7 (13) | 1 (2) |
| 21. Provide instruction on how to perform the behaviour | 4 (8) | 6 (11) | 1 (2) |
| 22. Model/demonstrate the behaviour | 2 (4) | 0 (0) | 0 (0) |
| 24. Environmental restructuring | 2 (4) | 0 (0) | 1 (2) |
| 32. Fear arousal | 0 (0) | 1 (1) | 1 (2) |
| 33. Prompt self-talk | 2 (4) | 0 (0) | 0 (0) |
| 38. Time management | 2 (4) | 5 (9) | 0 (0) |
| 40.Stimulate anticipation of future rewards | 1 (2) | 0 (0) | 2 (5) |
